# Preserving Laryngo‐Esophageal Function in Patients With Hypopharyngeal Cancer Treated With Radiotherapy: Predictive Factors and Long‐Term Outcomes

**DOI:** 10.1002/cam4.70374

**Published:** 2024-11-02

**Authors:** Aya Nakajima, Michio Yoshimura, Shinya Hiraoka, Ryota Nakashima, Yo Kishimoto, Koichi Omori, Takashi Mizowaki

**Affiliations:** ^1^ Department of Radiation Oncology and Image‐Applied Therapy, Graduate School of Medicine Kyoto University Kyoto Japan; ^2^ Department of Otolaryngology, Head and Neck Surgery, Graduate School of Medicine Kyoto University Kyoto Japan

**Keywords:** hypopharyngeal cancer, intensity‐modulated radiotherapy, laryngo‐esophageal dysfunction‐free survival, predictive factors, prognostic nutritional index

## Abstract

**Background:**

Functional outcomes after hypopharyngeal cancer (HPC) treatment have a significant effect on patients' quality of life and prognosis. This study aimed to identify the predictive factors associated with laryngo‐esophageal dysfunction in patients with HPC who received definitive radiotherapy.

**Methods:**

Patients with HPC treated with definitive intensity‐modulated radiotherapy between 2007 and 2019 at our institution were retrospectively evaluated. Laryngo‐esophageal dysfunction‐free survival (LDFS) events were defined as local recurrence, laryngo‐esophageal dysfunction (defined as tracheostomy or feeding tube dependence), or death from any cause.

**Results:**

The median follow‐up period was 61 months for the 80 patients included in the study. The 5‐year LDFS rate was 47%. A clinical T4 stage and lower pretreatment prognostic nutritional index (PNI) were independently associated with a lower LDFS.

**Conclusion:**

A clinical T4 stage and lower pretreatment PNI were identified as predictors of a lower LDFS after definitive radiotherapy for HPC.

## 1 Introduction

The incidence of hypopharyngeal cancer (HPC) is approximately 84,000 and 3000 cases per year worldwide and in Japan, respectively [1, 2]. In Japan, the incidence of HPC has been increasing in recent years [3], with more than half of the patients aged 70 years or older [2]. Prognosis of HPC is worse than that of other head and neck cancers because it is often diagnosed at an advanced stage owing to its asymptomatic nature in the early stages and high frequency of lymph node metastasis [4].

Although larynx‐preserving surgery is indicated for early‐stage disease, total laryngectomy with pharyngectomy is the standard surgical option for locally advanced HPC [4, 5]. Alternatively, definitive radiotherapy with or without concurrent and induction chemotherapy is widely used to preserve the larynx [6, 7]. Nevertheless, loss of laryngo‐esophageal function is not uncommon after radiotherapy because of local recurrence or late toxicities such as dysphagia, laryngeal edema, and laryngeal necrosis [8, 9, 10, 11]. Given that these laryngo‐esophageal dysfunction events may affect prognosis, predicting the possibility of preserving the laryngeal function prior to treatment is important for determining the treatment of choice. However, there is currently no established method to predict laryngo‐esophageal function preservation in patients with HPC before treatment. This study aimed to analyze long‐term oncological and functional outcomes in patients with HPC who received definitive radiotherapy and identify the predictive factors associated with laryngo‐esophageal dysfunction.

## 2 Materials and Methods

### 2.1 Study Design and Patient Population

We retrospectively evaluated patients with hypopharyngeal squamous cell carcinoma treated with curative‐intent intensity‐modulated radiotherapy (IMRT) between 2007 and 2019 at Kyoto University Hospital. Patients who underwent surgery for the primary tumor or those who discontinued radiotherapy after receiving less than 60 Gy were excluded. This study was conducted in accordance with the Declaration of Helsinki and the National Guidelines for Clinical Research and approved by the Institutional Review Board (approval number: R1048). Informed consent was not required due to the retrospective nature of the study; instead, an opt‐out method was applied according to national guidelines [12].

### 2.2 Treatment

Patient treatment recommendations were discussed by the weekly multidisciplinary head and neck tumor board, which included head and neck surgeons, radiation oncologists, and medical oncologists. Regarding radiotherapy, the patients were immobilized in the supine position with a thermoplastic head and shoulder mask. Contrast‐enhanced computed tomography (CT) images were taken from head to chest for treatment planning. Diagnostic magnetic resonance imaging (MRI) and/or fluorodeoxyglucose positron emission tomography (FDG‐PET) was fused to the planning CT scans to assist target delineation. The gross tumor volume (GTV) was defined as the primary tumor and metastatic lymph nodes identified on CT, MRI, FDG‐PET, and fiberoptic endoscopy. The clinical target volume (CTV) 70 was defined as the GTV plus a margin of 5–20 mm to cover subclinical microscopic diseases. CTV 63 included the entire hypopharyngeal and laryngeal mucosa and high‐risk lymph node area; CTV 56 included the bilateral elective lymph node areas. The planning target volume (PTV) was generated by adding a 5‐mm margin to the CTV. The simultaneous integrated boost technique was used to deliver 70, 63, and 56 Gy to PTV 70, PTV 63, and PTV 56, respectively, in 35 fractions with the aim to cover each PTV with 95% of the prescribed dose. IMRT was delivered using a 7‐field sliding window technique before May 2015, and volumetric modulated arc radiotherapy (VMAT) was administered thereafter. No dose constraints were applied to the dysphagia and aspiration‐related structures (DARS) in treatment plans for patients included in this study. To analyze the relationship between post‐irradiation swallowing function and doses to DARS, the superior, middle and inferior pharyngeal constrictor muscles (PCMs) were recontoured following the published contouring guidelines [13] by a radiation oncologist specializing in head and neck cancer (A.N.) in the treatment planning system. All treatment plans were subsequently recalculated; the median dose for PCMs was evaluated.

Concurrent chemotherapy was administered to all patients, except those in the early stages or those who were medically ineligible (e.g., > 80 years or with comorbidities). Induction chemotherapy was administered to patients with locally advanced disease. The triweekly cisplatin regimen was selected for concurrent chemotherapy, and the docetaxel–cisplatin–5‐FU or the cisplatin–5‐FU regimens were selected for induction chemotherapy. For hospitalized patients, swallowing and feeding assessments, and rehabilitation were performed by a multidisciplinary team consisting of head and neck surgeons, dentists, speech therapists, dietitians, nurses, pharmacists, and dental hygienists.

### 2.3 Follow‐Up

After the completion of radiotherapy, follow‐up observations, including physical and endoscopic examinations, were performed every month for the first year, every 2–3 months from the second to the fifth year, and every 6 months thereafter. Imaging tests with CT or MRI were performed every 3–6 months for the first 2 years and every 6–12 months from the third to the fifth year. The follow‐up period was defined as the initial day of radiotherapy.

### 2.4 Clinical Data Collection

Data on patients and disease‐specific variables, such as the history of habitual smoking and alcohol use, Adult Comorbidity Evaluation‐27 (ACE‐27) score [14], clinical stage, and tumor subsite, were retrospectively collected from medical records. Clinical stages were assessed according to the Union for International Cancer Control 7th edition. The pretreatment prognostic nutritional index (PNI), which was defined as 10 × serum albumin (g/dL) + 0.005 × peripheral lymphocyte count (/mm^3^) [15], and body mass index (BMI) were calculated from the latest data before initiating radiotherapy or chemotherapy.

### 2.5 Statistical Analysis

The laryngo‐esophageal dysfunction‐free survival (LDFS) rate was defined as survival with all of the following conditions: local control without any surgery (except biopsy) to the primary site, no permanent tracheostomy, and oral nutrition only [8, 16]. Events for LDFS were classified into 3 types: local recurrence, laryngo‐esophageal dysfunction (including total laryngectomy, permanent tracheostomy, and feeding tube‐ or gastrostomy‐dependent > 1 year), and death from any cause. The overall survival (OS), locoregional control (LRC), progression‐free survival (PFS), LDFS, and cumulative incidence of laryngo‐esophageal dysfunction were analyzed using the Kaplan–Meier method and were calculated from the initiation of radiotherapy until the date of event occurrence or last follow‐up. The log‐rank test was used to compare LDFS between the groups. Receiver operating characteristic curve analysis was performed to determine the optimal cutoff value for the PNI. For BMI and age, the median values were used as thresholds to divide the patients into two groups. Univariate and multivariate analyses were performed using a Cox proportional hazards model. PNI values and the mean dose to PCMs for each group of LDFS events are presented as the mean ± SD. Differences between the groups were evaluated using Student's *t*‐tests. All analyses were performed using R software (R Foundation for Statistical Computing, Vienna, Austria). Statistical significance was set at *p* ≤ 0.05.

## 3 Results

During the study period, 81 consecutive patients with HPC were treated with IMRT/VMAT with curative intent. One patient who discontinued radiotherapy at 52 Gy because of pneumonia was excluded from the study. In total, 80 patients were included in this study. The patient characteristics are presented in Table 1. The statistically significant cutoff value of the PNI was 48 (area under the curve, 0.66; 95% confidence interval [CI]: 0.53–0.79; *p* = 0.013) for LDFS. Systemic therapy was administered to 67 patients (induction chemotherapy plus concurrent chemotherapy for 35, concurrent chemotherapy alone for 30, and induction chemotherapy alone for 2). The median total radiotherapy dose was 70 Gy (range, 62–70 Gy).

**TABLE 1:** Patient characteristics.

	All patients (*N* = 80)
Age (years), median (range)	69 (40–84)
Male, *n* (%)	72 (90.0)
Subsite, *n* (%)	
Pyriform sinus	61 (76.3)
Posterior wall	12 (15.0)
Postcricoid	7 (8.7)
Clinical stage^a^, *n* (%)	
I	3 (3.8)
II	15 (18.7)
III	14 (17.5)
IV	48 (60.0)
Clinical T stage^a^, *n* (%)	
T1	10 (12.5)
T2	35 (43.8)
T3	20 (25.0)
T4	15 (18.7)
Clinical N stage^a^, *n* (%)	
N0	26 (32.5)
N1	10 (12.5)
N2	42 (52.5)
N3	2 (2.5)
BMI (kg/m^2^), median (range)	21.8 (14.0–30.8)
PNI, median (range)	47.5 (36.3–57.5)
Smoking ≥ 10 pack‐years, *n* (%)	65 (81.3)
Current alcohol consumption, *n* (%)	65 (81.3)
ACE‐27 index, *n* (%)	
0	16 (20.0)
1	28 (35.0)
2	14 (17.5)
3	22 (27.5)

Abbreviations: ACE‐27, Adult Comorbidity Evaluation‐27; BMI, body mass index; PNI, prognostic nutritional index.

^a^ Based on the Union for International Cancer Control 7th edition.

**FIGURE 1:**
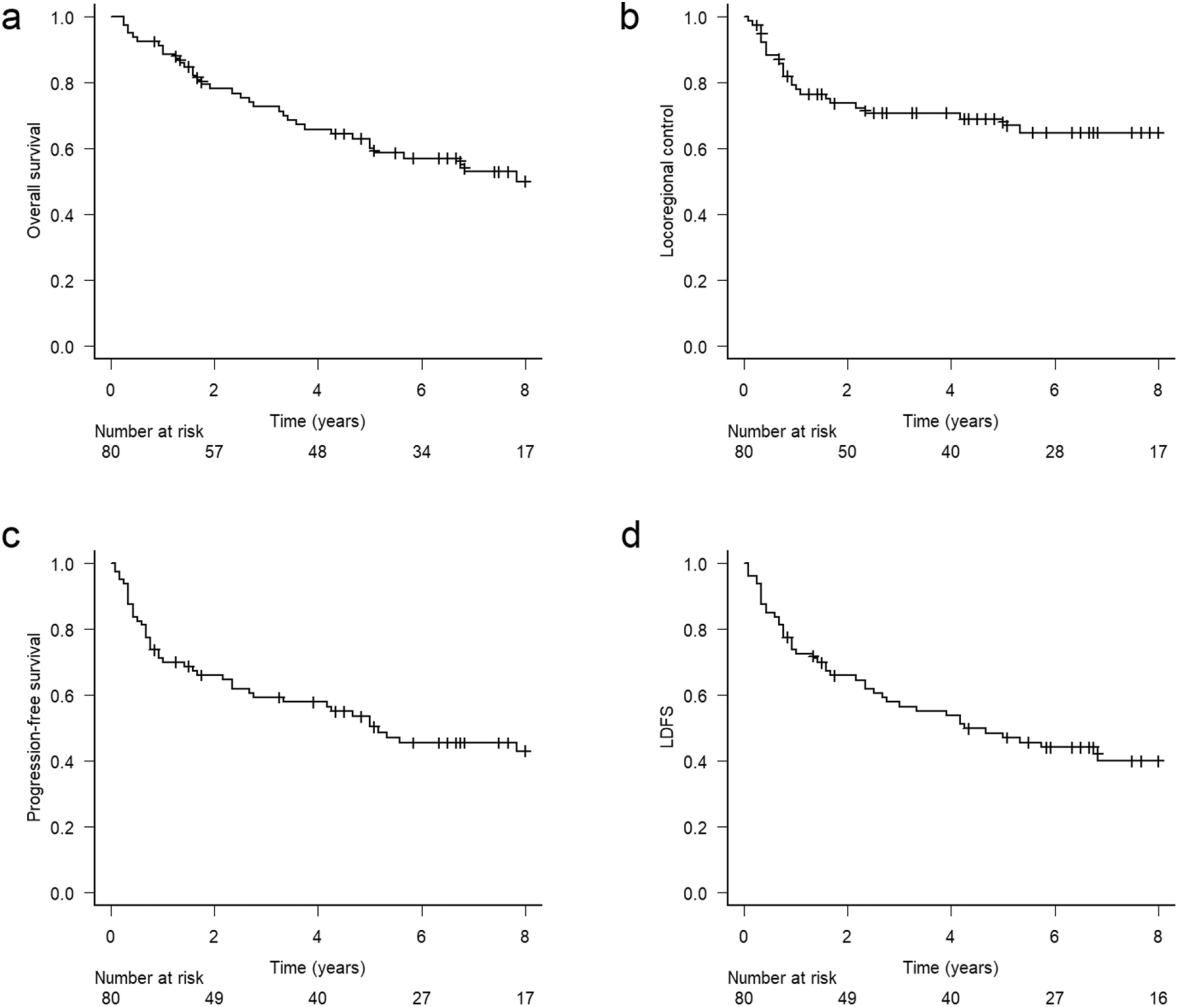
(a) Overall survival, (b) locoregional survival, (c) progression‐free survival, and (d) LDFS rates of all patients. LDFS, laryngo‐esophageal dysfunction‐free survival.

The median follow‐up period was 61 months (range, 3–170 months). The OS, LRC, PFS, and LDFS rates at 5 years were 60%, 67%, 50%, and 47%, respectively (Figure 1). LDFS events were observed in 49 (61%) patients (local recurrence in 23, laryngo‐esophageal dysfunction in 10, and death in 16). Among the 23 patients with local recurrence, two had regional recurrence before local recurrence, four had both local and regional recurrence, and 17 developed isolated local recurrence. Seven patients with local recurrence underwent salvage total laryngectomy; salvage was successful in four of them. One patient experienced a tracheoesophageal fistula, and two patients experienced delayed wound healing after total laryngectomy. Two patients were successfully treated with endoscopic surgery without complications.

Among the 10 patients with laryngeal dysfunction, five were dependent on tube feeding, three had permanent tracheostomies, one had total laryngectomy due to repeated aspiration, and 1 died from aspiration pneumonia. Prophylactic percutaneous gastrostomy tube placement was performed in 21 patients. During radiotherapy, 31 patients required gastrostomy or nasogastric tube feeding. At 1, 3, and 6 months after the completion of radiotherapy, 16, 11, and seven patients were dependent on tube feeding, respectively. Two patients were still on tube feeding at 1‐year post‐treatment and were counted as LDFS events. Three other patients required gastrostomy owing to pharyngeal stenosis or dysphagia for more than 3 years after treatment. Comparison of mean doses of PCMs between the patients with laryngeal dysfunction and those who survived with a functional larynx showed no significant differences in superior PCM (60.36 ± 2.65 vs. 58.09 ± 5.98, *p* = 0.25), middle PCM (69.28 ± 3.10 vs. 68.98 ± 3.14, *p* = 0.79), and inferior PCM (72.39 ± 1.57 vs. 71.98 ± 1.30, *p* = 0.41).

**FIGURE 2:**
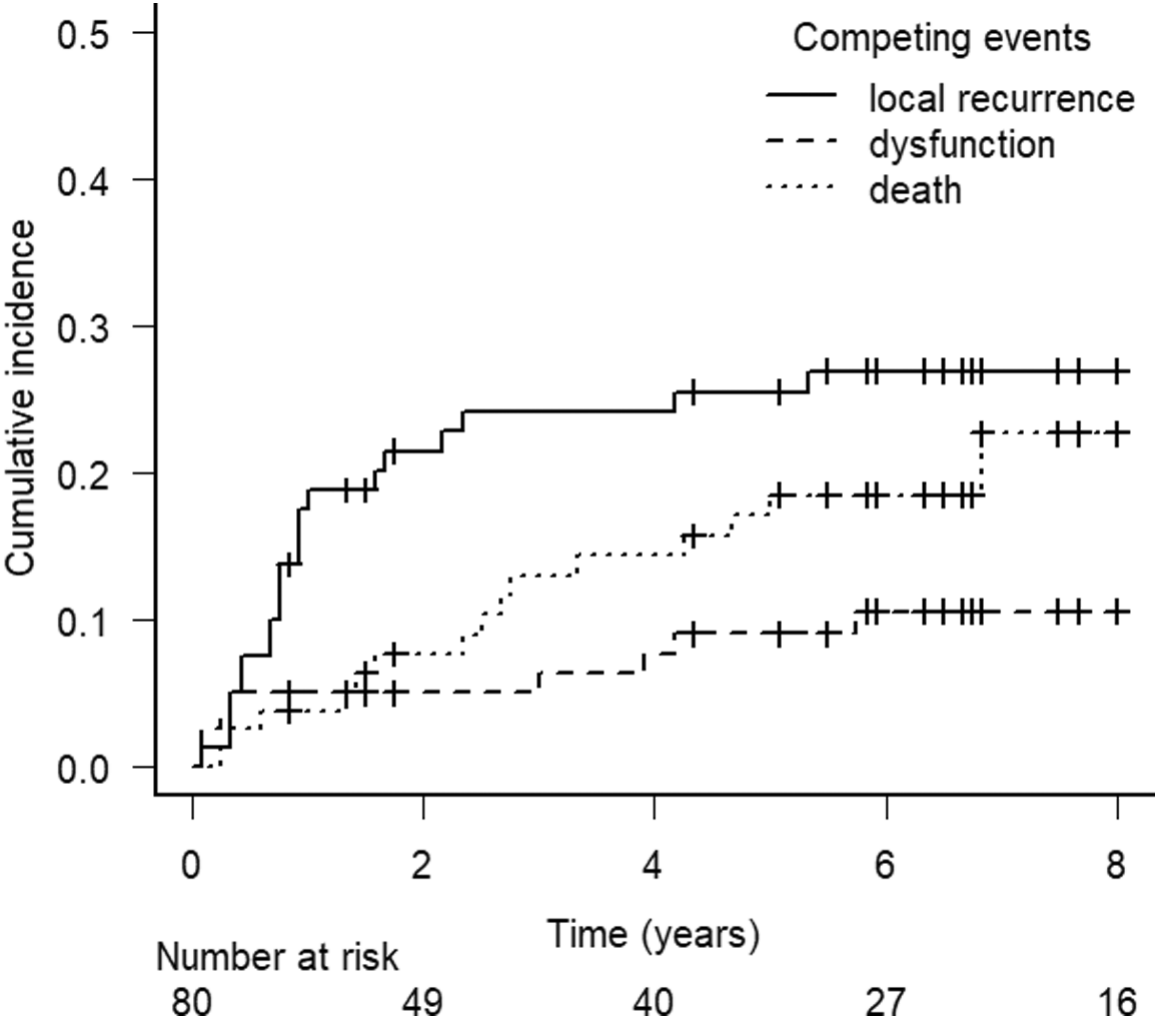
The cumulative incidence of LDFS events. LDFS, laryngo‐esophageal dysfunction‐free survival.

Of the patients wherein death was the first event, 7 died of distant metastasis from HPC without local recurrence or laryngeal dysfunction. Nine patients died from other causes, including six from other cancers. The cumulative incidence of LDFS events is shown in Figure 2. Most local recurrences occurred in the first 2 years, whereas death and laryngeal dysfunction were observed over a longer period.

**FIGURE 3:**
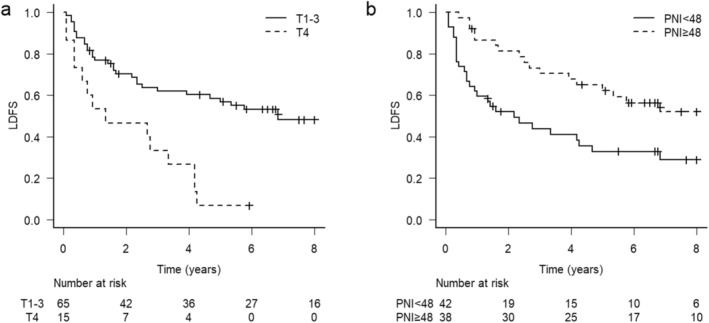
LDFS rates stratified by (a) T stage (T1–T3 vs. T4) and (b) PNI (< 48 vs. ≥ 48). LDFS, laryngo‐esophageal dysfunction‐free survival; PNI, prognostic nutritional index.

The univariate analysis of LDFS was conducted in relation to age, ACE‐27 scores, smoking history, alcohol status, clinical T stage, N stage, pretreatment BMI, PNI, and the use of induction chemotherapy. A clinical T4 stage (hazard ratio [HR]: 3.17; 95% CI: 1.61–5.97; *p* < 0.001) and lower pretreatment PNI (HR: 2.17; 95% CI: 1.23–3.94; *p* = 0.009) were significantly associated with a lower LDFS. The multivariate analysis revealed that both the T4 stage (HR: 1.94; 95% CI: 1.08–3.49; *p =* 0.027) and lower pretreatment PNI (HR: 2.80; 95% CI: 1.45–5.43; *p =* 0.002; Table 2) were independent prognostic factors for a worse LDFS. The LDFS rates stratified by T stage and pretreatment PNI are shown in Figure 3.

**TABLE 2:** Univariate and multivariate analyses for LDFS.

Variables		*n*	Univariate			Multivariate		
			HR	95% CI	*p*	HR	95% CI	*p*
Age	< 69	40	1		NA	NA	NA	NA
	≥ 70	40	1.12	0.64–1.97	0.69	NA	NA	NA
T stage	≤ 3	65	1		NA	1	NA	NA
	4	15	3.17	1.61–5.97	**< 0.001**	2.80	1.41–5.34	**0.002**
N stage	0–1	36	1		NA	NA	NA	NA
	2–3	44	1.71	0.97–3.13	0.07	NA	NA	NA
BMI	< 22.0	40	1.38	0.79–2.46	0.26	NA	NA	NA
	≥ 22.0	40	1		NA	NA	NA	NA
PNI	< 48	42	2.17	1.23–3.94	**0.009**	1.95	1.09–3.54	**0.027**
	≥ 48	38	1		NA	1	NA	NA
Smoking	≥ 10 pack‐years	65	1.68	0.77–4.42	0.23	NA	NA	NA
	< 10 pack‐years	15	1		NA	NA	NA	NA
Alcohol	Current	65	1.43	0.68–3.49	0.39	NA	NA	NA
	Former/never	15	1		NA	NA	NA	NA
ACE‐27	0–1	44	1		NA	NA	NA	NA
	2–3	36	1.43	0.80–2.53	0.22	NA	NA	NA
ICT	Yes	37	0.88	0.50–1.55	0.67	NA	NA	NA
	No	43	1		NA	NA	NA	NA

Abbreviations: ACE‐27, Adult Comorbidity Evaluation‐27; BMI, body mass index; CI, confidence interval; HR, hazards ratio; ICT, induction chemotherapy; LDFS, laryngo‐esophageal dysfunction‐free survival; NA, not applicable; PNI, prognostic nutritional index. Bold values indicate *p*‐values < 0.05, which we consider statistically significant.

**FIGURE 4:**
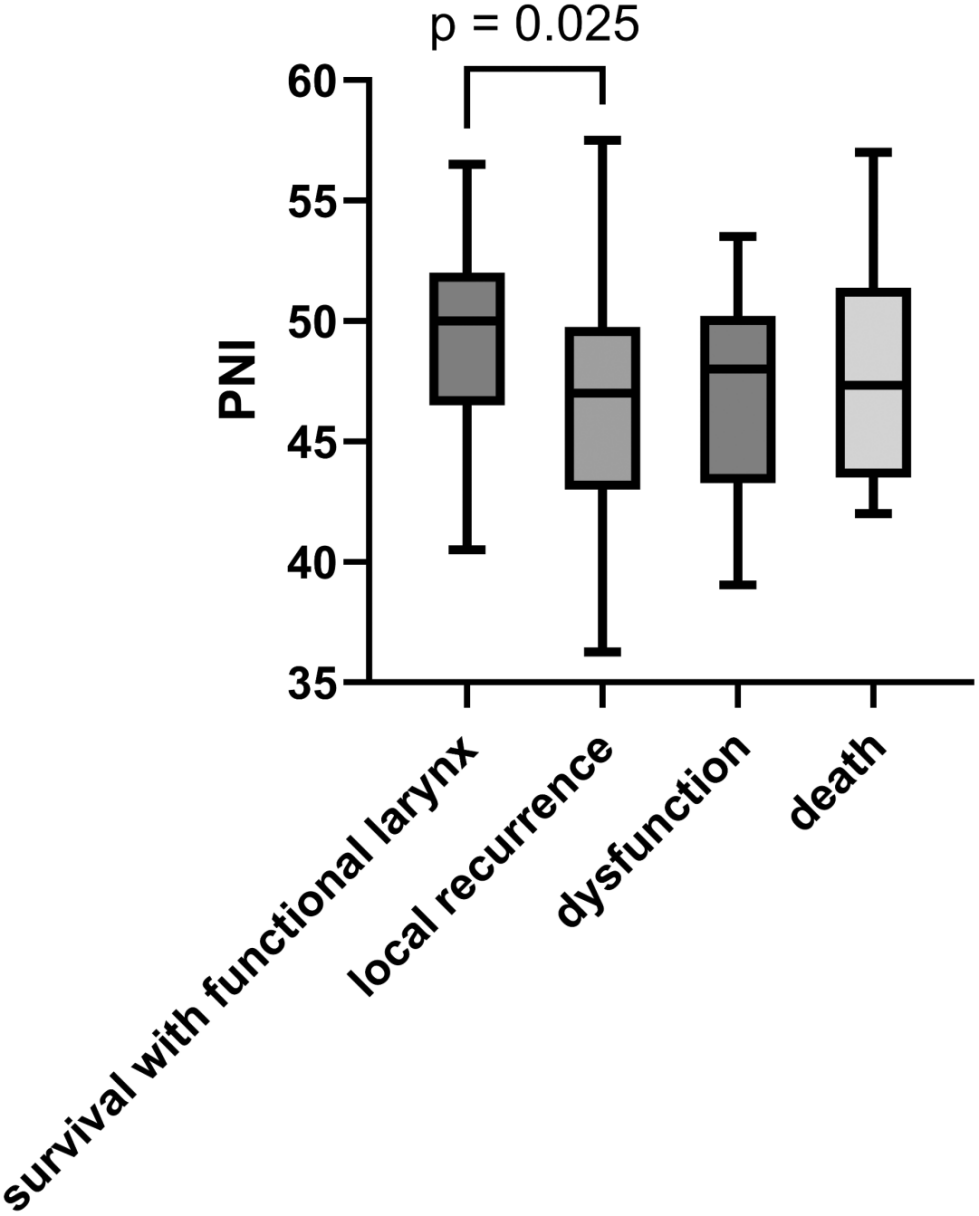
Box‐plot diagram of PNI for each LDFS event. PNI, prognostic nutritional index; LDFS, laryngo‐esophageal dysfunction‐free survival.

Comparing the PNI values for each group of LDFS events, patients with local recurrence had a significantly lower PNI (46.56 ± 5.30, *p =* 0.025) than patients who survived with a functional larynx (49.63 ± 4.07). There was a trend toward a lower PNI in patients with laryngeal dysfunction (47.23 ± 4.53) or death (47.57 ± 4.35) compared with that in patients who survived with a functional larynx, but the difference was not statistically significant (Figure 4).

## 4 Discussion

We conducted an in‐depth analysis of the long‐term oncologic and functional outcomes in patients with HPC who received IMRT/VMAT treatment with a median follow‐up of 61 months. Although several single‐center retrospective analyses of IMRT outcomes for HPC have been published [10, 17, 18, 19], this study represents the longest follow‐up, to our best knowledge. The study findings revealed that the T4 stage and PNI were significant prognostic factors for LDFS in patients with HPC treated with contemporary radiotherapy.

The goal of definitive radiotherapy for HPC is survival with disease control and the preservation of laryngeal and esophageal functions. Speech and swallowing have a significant impact on a patient's quality of life, and dysphagia can sometimes lead to reduced survival because of aspiration [20, 21]. Therefore, LDFS is considered an important endpoint of definitive radiotherapy for HPC [22]. Petersen et al. [16] conducted a retrospective analysis of the outcomes of definitive therapy for T1–T4 HPC and reported that the 5‐year LDFS rates after chemoradiotherapy and radiotherapy were 32% and 30%, respectively. Bozec et al. [23] reported a 2‐year LDFS rate of 58% in a retrospective analysis of 53 patients with T3–4 HPC undergoing an induction chemotherapy‐based larynx preservation program. Katsoulakis et al. [18] reported a 5‐year functional larynx preservation rate of 31.8% in patients with HPC treated with three‐dimensional conformal radiotherapy or IMRT. In the present study, the LDFS was 66% at 2 years and 47% at 5 years, which was slightly better than that reported in previous studies. This may be related to the fact that T1–T2 cases were included in our study and all patients were treated with IMRT/VMAT, whereas most previous reports on LDFS were based on conventional RT results. The median age of the patients in this study was 69 years, which was older than that in previous reports (58–63 years) [16, 18, 23]. Although age was not significantly associated with LDFS in this analysis, we believe that careful post‐treatment swallowing management is important given the large number of elderly patients with HPC. Moreover, a recent multicenter phase III randomized trial has reported that reducing the dose to PCMs with dysphagia‐optimized IMRT (DO‐IMRT) resulted in improved swallowing function compared with standard IMRT in patients with HPC and oropharyngeal cancer [24]. Given that DO‐IMRT was not administered in the present study and all patients uniformly received high doses to PCMs, we posit that there was no significant difference in the mean dose to PCMs between the patients who developed dysphagia or aspiration and those who did not.

T4 stage was reported to be the main poor prognostic factor for oncologic and functional outcomes [23]. In our institution, we predominantly recommended radical surgery in patients with T4 disease. However, we also perform radiotherapy treatment when patients refuse total laryngectomy or are not amenable to surgery owing to comorbidities. In the present study, the LDFS of patients with T4 disease was notably diminished. This finding supports the recommendation that radical surgery should be considered the primary treatment option for T4 disease [4, 7].

This is the first study to identify the PNI as a factor associated with LDFS in patients with HPC. The PNI serves as an indicator of a patient's nutritional and immune status and is often used to assess prognosis. Although the PNI was originally reported as a predictor of postoperative complications in gastrointestinal surgery [15], several studies have suggested a correlation between the PNI and the prognosis of patients with cancer [25, 26]. In head and neck cancer, an association between the PNI and OS, PFS, disease‐specific survival, and distant metastasis‐free survival has been reported in patients undergoing radiotherapy or surgery [25, 27, 28]. Furthermore, the PNI has been reported to be a predictor of radiotherapy toxicities, such as mucositis and weight loss, in head and neck cancer [29]. LDFS is an endpoint that reflects both the prognosis (local recurrence and survival) and toxicity (functional impairment), and the PNI is considered a suitable indicator for LDFS.

However, our study has some limitations. In previous studies including patients with head and neck cancer, the cutoff values for the PNI varied from 40 to 57 [25, 27, 28]. In the present study, the cutoff value was 48, which is within the range of previous studies. Nevertheless, it is necessary to consider the possibility that there may be differences in the optimal cutoff value for the PNI between primary sites and institutions for clinical applications. Furthermore, analysis of PNI values in each group of LDFS events showed that patients with local recurrence had significantly lower PNI than did those who survived with a functional larynx. However, no significant difference was observed in patients with laryngeal dysfunction or death. Since local recurrence accounts for nearly half of all LDFS events, we cannot rule out the possibility that PNI is actually a predictor of local recurrence rather than LDFS. Another limitation of this study is its single‐center retrospective nature. A large prospective study is needed to assess the validity of the PNI as a predictor of LDFS.

In conclusion, a lower pretreatment PNI and clinical T4 stage were significant predictors of a worse LDFS after definitive radiotherapy for HPC, with low PNI correlating strongly with local recurrence; hence, an assessment of the pretreatment PNI may help determine treatment strategies for HPC.

## Author Contributions

**Aya Nakajima:** conceptualization (lead), data curation (lead), formal analysis (lead), funding acquisition (lead), investigation (lead), methodology (lead), project administration (equal), writing – original draft (lead). **Michio Yoshimura:** conceptualization (supporting), investigation (supporting), writing – review and editing (equal). **Shinya Hiraoka:** conceptualization (supporting), investigation (supporting), writing – review and editing (equal). **Ryota Nakashima:** conceptualization (supporting), investigation (supporting), writing – review and editing (equal). **Yo Kishimoto:** investigation (supporting), writing – review and editing (equal). **Koichi Omori:** investigation (supporting), writing – review and editing (equal). **Takashi Mizowaki:** conceptualization (supporting), investigation (supporting), resources (lead), supervision (lead), writing – review and editing (equal).

## Ethics Statement

The study design was approved by the Ethics Committee of Kyoto University Graduate School and Faculty of Medicine and conducted in accordance with the Declaration of Helsinki and the National Guidelines for Clinical Research.

## Consent

The requirement for informed consent was waived because of the retrospective nature of the study; instead, an opt‐out method was applied.

## Conflicts of Interest

The authors declare no conflicts of interest.

## Data Availability

Data that support the findings of this study are not openly available because of reasons of sensitivity. However, the data are available from the corresponding author upon reasonable request.
